# Supplementary material for: The role of age in choosing high-efficacy treatment for multiple sclerosis: an Austrian MS Database study
Source: J Neurol. 2026 Apr 9;273(5):262. doi: 10.1007/s00415-026-13787-0 (PMC13065530; doi:10.1007/s00415-026-13787-0)
Supplement: Supplementary file 1 — Supplementary file1 (PDF 326 KB) [file 415_2026_13787_MOESM1_ESM.pdf]

Supplemental Table 1: Different disease-modifying treatments applied in the study cohort

	**Total cohort**	**Relapse activity**	**No relapse activity**
**Moderate-efficacy DMT**	159 (74)	66 (81)	93 (69)
Interferon-beta	53 (25)	23 (28)	30 (22)
Glatiramer acetate	31 (14)	14 (17)	17 (13)
Dimethyl fumarate	68 (32)	25 (31)	43 (32)
Teriflunomide	7 (3)	4 (5)	3 (2)
**High-efficacy DMT**	56 (26)	15 (19)	41 (31)
Fingolimod	9 (4)	4 (5)	5 (4)
Ozanimod	4 (2)	0 (0)	4 (3)
Cladribine	9 (4)	3 (4)	6 (4)
Natalizumab	6 (3)	1 (1)	5 (4)
Rituximab	20 (9)	5 (6)	15 (11)
Ocrelizumab	5 (2)	1 (1)	4 (3)
Ofatumumab	1 (0.5)	0 (0)	1 (1)
Alemtuzumab	2 (2)	1 (1)	1 (1)

Legend:

Data are given as n (%).

Abbreviations: DMT = disease-modifying treatment

Supplemental Table 2: Demographic, clinical and imaging characteristics per center

	**Graz**	**Innsbruck**	**Vienna**
**Number of patients**	64	78	73
**Age (years)**	33 (27-41)	37 (32-44)	55 (49-60)
**Sex (female)**	52 (80)	47 (60)	43 (60)
**Disease duration (months)**	3 (1-7)	2 (1-5)	2 (1-8)
**EDSS at DMT start**	1 (0-2)	1 (0-1.5)	2 (1-2.5)
**≥9 T2 lesions at DMT start**	30 (46)	42 (54)	52 (72)
**≥1 CEL**	24 (38)	30 (38)	17 (23)
**OCB positivity[1]**	57 (92)	70 (99)	58 (97)
**Number of relapses 12 months prior DMT start**	1 (1-1)	1 (1-1)	1 (1-1)

Legend:

Data are given as median (25th-75th percentile) and n (%).

[1] Results of OCB available of 62 patients in Graz, of 71 in Innsbruck and 60 patients in Vienna.

Abbreviations: CEL = contrast-enhancing lesion, DMT = disease modifying treatment, EDSS = Expanded Disability Status Scale, OCB = oligoclonal bands.

Supplemental Table 3: Baseline characteristics according to different age

	Total cohort	Age ≤30	30<Age≤40	40<Age≤50	Age >50
**Number of patients**	215	40	66	47	62
**Sex (female)**	142 (66)	30 (75)	48 (73)	25 (53)	39 (63)
**Disease duration (months)**	2 (1-7)	1 (1-5)	3 (1-6)	3 (1-8)	3 (1-10)
**EDSS at DMT start**	1 (0-2)	1 (0-2)	1 (0-2)	1 (0-2)	2 (1-3)
**≥9 T2 lesions at DMT start**	124 (58)	21 (53)	30 (46)	25 (53)	48 (77)
**≥1 CEL**	71 (33)	15 (38)	26 (39)	14 (30)	16 (26)
**OCB positivity**[1]	185 (96)	38 (97)	60 (97)	39 (95)	48 (94)
**Number of relapses 12 months prior DMT start**	1 (1-1)	1 (1-1)	1 (1-1)	1 (1-1)	1 (1-1)
**Moderate-efficacy DMT**	159 (74)	29 (73)	56 (85)	39 (83)	57 (92)
**High-efficacy DMT**	56 (26)	11 (28)	10 (15)	8 (17)	5 (8)

Legend:

Data are given as median (25th-75th percentile) and n (%).

[1] Results of OCB available of 193 patients.

Abbreviations: CEL = contrast-enhancing lesion, DMT = disease modifying treatment, EDSS = Expanded Disability Status Scale, OCB = oligoclonal bands.

Supplemental Table 4: Demographic, clinical and imaging characteristics

	Total cohort	Disability accrual	No disability accrual	p-value
**Number of patients**	215	46	169	
**Age (years)**	41 (32-53)	46 (37-55)	38 (32-52)	0.002§
**Sex (female)**	142 (66)	32 (70)	110 (65)	0.694#
**Disease duration (months)**	2 (1-7)	3 (1-9)	2 (1-6)	0.526§
**EDSS at DMT start**	1 (0-2)	1 (0-2)	1 (0-2)	0.711§
**≥9 T2 lesions at DMT start**	124 (58)	29 (63)	95 (56)	0.507#
**≥1 CEL**	71 (33)	15 (33)	56 (33)	1.000*
**OCB positivity[1]**	185 (96)	39 (93)	146 (97)	0.507#
**Number of relapses 12 months prior DMT start**	1 (1-1)	1 (1-1)	1 (1-1)	0.510§
**Moderate-efficacy DMT**	159 (74)	35 (76)	124 (73)	0.855[2]
**High-efficacy DMT**	56 (26)	11 (24)	45 (27)	

Legend:

Data are given as median (25th-75th percentile) and n (%). Comparisons were done by #χ^2 test, *Fisher's exact test and §Mann-Whitney U test.

[1] Results of OCB available of 193 patients (39 in the disability accrual group, 146 in the non-disability accrual group)

[2] Frequency of moderate vs. high-efficacy DMT in patients with and without disability accrual were compared.

Abbreviations: CEL = contrast-enhancing lesion, DMT = disease modifying treatment, EDSS = Expanded Disability Status Scale, OCB = oligoclonal bands.

Supplemental Table 5: Cox regression predicting the risk of relapse dependent on disease-modifying treatment and age in the subgroup of CD20 monoclonal antibody versus M-DMT

	**Coefficient**	**Standard Error**	**P value**	**Hazard Ratio**	**95%-CI**	
**DMT** (ref: M-DMT)	-4.099	1.315	0.002	0.017	0.001	0.218
**Age** (years) **at DMT start**	-0.052	0.012	<0.001	0.949	0.926	0.972
**Sex** (ref: male)	0.435	0.294	0.138	1.545	0.869	2.748
**EDSS at DMT start**	0.155	0.111	0.162	1.167	0.940	1.450
**Disease duration** (months) **at DMT start**	0.001	0.004	0.898	1.001	0.992	1.008
**Number of relapses 12 months prior to DMT start**	0.092	0.230	0.688	1.097	0.699	1.720
**T2 MRI lesions at DMT start** (ref: <9)	0.444	0.267	0.097	1.559	0.923	2.631
**CEL MRI lesions at DMT start** (ref: 0)	0.136	0.265	0.609	1.145	0.681	1.927
**H-DMT : Age**	0.080	0.030	0.008	1.083	1.021	1.149

Cox Snell pseudo-R^2: 0.32

Legend:

Abbreviations: CEL, contrast-enhancing lesions, CI = confidence interval, DMT = disease modifying treatment, EDSS = Expanded Disability Status Scale, H-DMT = high-efficacy disease modifying treatment, M-DMT = moderate-efficacy disease modifying treatment, MRI = magnetic resonance imaging, ref = reference category

Supplemental Table 6: Determination of age when superiority of H-DMT over M-DMT is vanished

Equation

h(t | DMT, age, ...) = -2.904 · DMT - 0.048 · Age + 0.056 · Age:DMT + …

Scenario 1: High-efficacy DMT

h(t | DMT=1, age, ...) = -2.904 - 0.048 · Age + 0.056 · Age + …

Scenario 2: Moderate-efficacy DMT

h(t | DMT=0, age, ...) = -0.048 · Age + …

Determination of age when superiority of H-DMT over M-DMT is vanished

h(t | DMT=1, age, ...) = h(t | DMT=0, age, ...)

2.904 = 0.056 · Age

Age = 51.86 years

Legend:

h(t | DMT, age, ...) = Hazard rate at time *t* for an individual with covariates DMT, age, …

Supplemental Table 7: Computed probabilities for freedom of relapse depending on age, type of DMT and disease activity

**Age** (years)	**Computed probabilities of relapse freedom in patients with**				**Difference in computed probabilities between patients with low and high disease activity**	
	***low* disease activity**		***high* disease activity**			
	**M-DMT**	**H-DMT**	**M-DMT**	**H-DMT**	**M-DMT**	**H-DMT**
60	0.95	0.95	0.80	0.80	0.15	0.15
50	0.94	0.95	0.76	0.78	0.19	0.17
40	0.91	0.95	0.64	0.79	0.27	0.16
30	0.86	0.95	0.48	0.81	0.37	0.15
20	0.78	0.96	0.31	0.82	0.47	0.14

Legend:

The computed probabilities for freedom of relapse were derived from the Cox regression model (Table 2) depending on the type of DMT (M-DMT vs. H-DMT) for different patients' ages (20, 30, 40, 50, 60) and for different baseline MS disease activity. For low disease activity, co-variables were set as follows: T2L to "<9", CEL to "0", number of relapses 12 months prior to DMT start = 0, EDSS score at DMT start = 0, disease duration in months = 1 and sex to "male". For high disease activity, T2L was set to " ≥9", CEL to "1", number of relapses 12 months prior to DMT start = 2, EDSS score at DMT start = 4, disease duration in months = 12 and sex to "female".

Abbreviations: CEL = contrast-enhancing lesion. EDSS = Expanded Disability Status Scale. M-DMT = moderate-efficacy disease-modifying treatment. H-DMT = moderate-efficacy disease-modifying treatment. T2L = number of hyperintense lesions on T2-weighted MRI

Supplemental Table 8: Cox regression predicting the risk of disability accrual

	Coefficient	Standard Error	P value	Hazard Ratio	95%-CI	
**DMT** (ref: M-DMT)_	-1.947	1.398	0.164	0.143	0.009	2.210
**Age** (years)	0.004	0.017	0.808	1.004	0.971	1.038
**Sex** (ref: male)	0.241	0.334	0.471	1.272	0.661	2.447
**EDSS at DMT start**	0.020	0.127	0.873	1.021	0.795	1.310
**Disease duration** (months)	0.002	0.005	0.680	1.002	0.993	1.011
**Number of relapses 12 months prior to DMT start**	0.423	0.297	0.155	1.527	0.853	2.735
**T2 lesions at DMT start** (ref: <9)	-0.115	0.338	0.734	0.892	0.460	1.729
**CEL at DMT start** (ref: 0)	-0.170	0.342	0.619	0.844	0.431	1.649
**H-DMT : Age**	0.047	0.030	0.119	1.048	0.988	1.112

Cox Snell pseudo-R^2: 0.16

Legend:

Abbreviations: CEL, contrast-enhancing lesions, CI = confidence interval, DMT = disease modifying treatment, EDSS = Expanded Disability Status Scale, H-DMT = high-efficacy disease modifying treatment, M-DMT = moderate-efficacy disease modifying treatment, ref = reference category

Supplemental Table 9: Cox regression predicting the risk of progression independent of relapse activity

	Coefficient	Standard Error	P value	Hazard Ratio	95%-CI	
**DMT** (ref: M-DMT)_	-1.518	1.722	0.378	0.219	0.007	6.412
**Age** (years)	-0.003	0.021	0.904	0.997	0.957	1.039
**Sex** (ref: male)	-0.107	0.387	0.782	0.894	0.421	1.918
**EDSS at DMT start**	0.089	0.148	0.545	1.094	0.819	1.461
**Disease duration** (months)	0.002	0.005	0.614	1.002	0.993	1.012
**Number of relapses 12 months prior to DMT start**	0.019	0.370	0.959	1.019	0.493	2.105
**T2 lesions at DMT start** (ref: <9)	0.117	0.428	0.785	1.124	0.486	2.598
**CEL at DMT start** (ref: 0)	-0.249	0.418	0.551	0.780	0.344	1.768
**H-DMT : Age**	0.034	0.038	0.893	1.034	0.961	1.113

Cox Snell pseudo-R^2: 0.08

Legend:

Abbreviations: CEL, contrast-enhancing lesions, CI = confidence interval, DMT = disease modifying treatment, EDSS = Expanded Disability Status Scale, H-DMT = high-efficacy disease modifying treatment, M-DMT = moderate-efficacy disease modifying treatment, ref = reference category
